# Analysis of the Germination of Individual *Clostridium sporogenes* Spores with and without Germinant Receptors and Cortex-Lytic Enzymes

**DOI:** 10.3389/fmicb.2017.02047

**Published:** 2017-10-25

**Authors:** Shiwei Wang, Jason Brunt, Michael W. Peck, Peter Setlow, Yong-Qing Li

**Affiliations:** ^1^School of Chemical Engineering and Energy Technology, Dongguan University of Technology, Dongguan, China; ^2^Gut Health and Food Safety, Quadram Institute Bioscience, Norwich, United Kingdom; ^3^Department of Molecular Biology and Biophysics, University of Connecticut Health Center, Farmington, CT, United States; ^4^School of Electronic Engineering, Dongguan University of Technology, Dongguan, China; ^5^Department of Physics, East Carolina University, Greenville, NC, United States

**Keywords:** *Clostridium sporogenes*, spore germination, CaDPA release, commitment, memory

## Abstract

The Gram-positive spore-forming anaerobe *Clostridium sporogenes* is a significant cause of food spoilage, and it is also used as a surrogate for *C. botulinum* spores for testing the efficacy of commercial sterilization. *C. sporogenes* spores have also been proposed as a vector to deliver drugs to tumor cells for cancer treatments. Such an application of *C. sporogenes* spores requires their germination and return to life. In this study, Raman spectroscopy and differential interference contrast (DIC) microscopy were used to analyze the germination kinetics of multiple individual *C. sporogenes* wild-type and germination mutant spores. Most individual *C. sporogenes* spores germinated with L-alanine began slow leakage of ∼5% of their large Ca-dipicolinic acid (CaDPA) depot at *T*_1_, all transitioned to rapid CaDPA release at *T*_lag1_, completed CaDPA release at *T*_release_, and finished peptidoglycan cortex hydrolysis at *T*_lys_. *T*_1_, *T*_lag1_, *T*_release_, and *T*_lys_ times for individual spores were heterogeneous, but Δ*T*_release_ (*T*_release_ – *T*_lag1_) periods were relatively constant. However, variability in *T*_1_ (or *T*_lag1_) times appeared to be the major reason for the heterogeneity between individual spores in their germination times. After *T*_release_, some spores also displayed another lag in rate of change in DIC image intensity before the start of a second obvious DIC image intensity decline of 25–30% at *T*_lag2_ prior to *T*_lys_. This has not been seen with spores of other species. Almost all *C. sporogenes* spores lacking the cortex-lytic enzyme (CLE) CwlJ spores exhibited a *T*_lag2_ in L-alanine germination. Sublethal heat treatment potentiated *C. sporogenes* spore germination with L-alanine, primarily by shortening T_1_ times. Spores without the CLEs SleB or CwlJ exhibited greatly slowed germination with L-alanine, but spores lacking all germinant receptor proteins did not germinate with L-alanine. The absence of these various germination proteins also decreased but did not abolish germination with the non-GR-dependent germinants dodecylamine and CaDPA, but spores without CwlJ exhibited no germination with CaDPA. Finally, *C. sporogenes* spores displayed commitment in germination, but memory in GR-dependent germination was small, and less than the memory in *Bacillus* spore germination.

## Introduction

*Clostridium sporogenes* is a Gram-positive, spore-forming, anaerobic bacterium and a significant agent of food spoilage, although, unlike its close relative *C. botulinum, C. sporogenes* does not produce the neurotoxins responsible for botulism, a severe and fatal neuro-paralytic disease of humans and animals (Brown et al., 2012; Taylor et al., 2013). Due to its physiological and phylogenetical similarity to Group I *C. botulinum, C. sporogenes* spores are used as a surrogate for *C. botulinum* for testing the efficacy of sterilization regimens (Taylor et al., 2013). *C. sporogenes* spores are being investigated as vectors to deliver cancer-treating drugs to patients with tumor cells, where a nominal oxygen concentration enables spores to germinate, outgrow and locally produce the cancer drug (Nuyts et al., 2002). All applied uses of *C. sporogenes* spores take advantage of their two major features. First is their extreme high resistance, as like spores of many *Clostridium* species, *C. sporogenes* forms spores of extremely high heat resistance, and in the canning industry the ‘botulinum cook’ has been adopted as the standard minimum heat treatment (121°C for 3 min) for low acid canned foods (Peck, 2009); hence the use of *C. sporogenes* spores as a surrogate for *C. botulinum* spores. Second is the return to life of *C. sporogenes* spores in germination, essential for drug delivery by such spores, which can germinate and most importantly outgrow in tumor cell environments (Nuyts et al., 2002). Spore germination is also essential for *C. botulinum* spores to cause botulism, and bioinformatics analyses strongly suggest that *C. sporogenes* spore germination is very similar to that of spores of Group I *C. botulinum* (see below). Thus, understanding mechanisms of *C. sporogenes* spore germination may have practical applications in the management of *Clostridium* contamination and also lead to the development of new drug vectors. This knowledge may also lead to new methods for preventing spore germination and thereby subsequent growth, or efficiently promoting spore germination to facilitate inactivation of emergent sensitive vegetative cells or activation of desired drugs under appropriate conditions.

In recent years, much knowledge has been obtained about the germination of spores of Clostridiales species, such as spores of *C. perfringens* and *Clostridioides difficile*; however, most detailed knowledge about spore germination has come from studies on *Bacillus subtilis* spores (Setlow, 2006, 2013, 2014; Paredes-Sabja et al., 2011; Setlow et al., 2017). *B. subtilis* spore germination can be triggered by many different factors, including nutrients, enzymes, hydrostatic pressure, cationic surfactants such as dodecylamine, and a 1:1 chelate of Ca^2+^ and pyridine-2,6-dicarboxylic acid (dipicolinic acid; DPA) (CaDPA) (Setlow, 2003, 2013). Nutrient germinants for spores of *Bacillus* species include L-amino acids, purine nucleosides, and D-glucose as well as mixtures of such compounds. These compounds trigger germination by interacting with germinant receptors (GRs) present in spores’ inner membrane (IM), leading to a series of events taking place in a defined order (Setlow and Johnson, 2007; Yi and Setlow, 2010; Setlow, 2013; Luu and Setlow, 2014; Setlow et al., 2017). Initially, exposure of spores to nutrient germinants causes a reaction that commits spores to germinate even if the germinant is removed or displaced from its cognate GRs (Yi and Setlow, 2010; Setlow, 2013). This commitment step is followed by fast release of monovalent cations, as well as slow release of 10–20% of the spore core’s large pool (∼25% of core dry weight) of CaDPA (Wang et al., 2015b). Subsequently fast CaDPA release begins and release of all remaining CaDPA is a hallmark of the completion of stage I of germination (Setlow et al., 2017). The stage I events trigger spore entry into stage II when spores’ peptidoglycan (PG) cortex will be degraded by either of two redundant cortex-lytic enzymes (CLEs) in the cortex of spores, CwlJ and SleB; subsequently the spore core swells and takes up water, leading to initiation of metabolism and converting the germinated spore into a growing cell (Setlow, 2003; Paredes-Sabja et al., 2009; Li et al., 2013; Setlow et al., 2017).

Analysis of single spores’ image through phase-contrast or differential interference contrast (DIC) microscopy, as well as Raman spectroscopy, has divided the process of germination into four phases with the different phases ending at times *T*_1_, *T*_lag_, *T*_release_, and *T*_lys_ (Kong et al., 2011; Wang et al., 2015b; Setlow et al., 2017). *T*_1_ is the time when slow CaDPA leakage begins after germinant addition at *T*_0_ and is probably coincident with the time of commitment, between *T*_0_ and *T*_1_ there is no visible change in spores’ Raman spectra or DIC image intensity; *T*_lag_ is the time when the initiation of very rapid CaDPA release begins after the start of slow CaDPA leakage; *T*_release_ is the time for completion of rapid CaDPA release; following *T*_release_, spore refractility further declines somewhat due to cortex hydrolysis and core swelling, and the time when spore refractility becomes relatively constant is termed *T*_lys_. Notably, there are huge differences between the *T*_1_ times, in particular, for individual spores in spore populations, reflected in the heterogeneity in the germination of individuals in spore populations (Zhang et al., 2010a; Setlow et al., 2017).

While there are some similarities between *B. subtilis* and *Clostridium* spore germination, there are still a number of notable differences (Olguin-Araneda et al., 2015; Setlow et al., 2017). In particular, spores of *C. difficile* do not contain IM GRs, and spores of *C. difficile* and *C. perfringens* do not contain the CLEs CwlJ and SleB. Rather the latter spores contain the CLE SleC present in spores as an inactive zymogen, pro-SleC, which is activated by proteolytic cleavage early in germination. In contrast, *C. sporogenes* and Group I *C. botulinum* spores contain CwlJ and SleB, and at least SleB is needed for optimal germination and viability of Group I *C. botulinum* spores (Meaney et al., 2015; Brunt et al., 2016). Spores of these two *Clostridium* species also contain IM GRs.

In the current work laser tweezers Raman spectroscopy (LTRS) and DIC microscopy were used to analyze the kinetics of the germination of multiple individual C. *sporogenes* spores with different germinants, including a nutrient germinant, L-alanine, and the GR-independent germinants CaDPA and dodecylamine. The roles of various germination proteins, including GRs and individual CLEs in C. *sporogenes* spore germination were also investigated, as well as the effects of sublethal heat shock prior to germination, and spore germination commitment and memory. The results of this study provide new insight into the germination of C. *sporogenes* spores, and thus likely that of Group I *C. botulinum* spores.

## Materials and Methods

### *Clostridium* Strains, Growth Conditions and Spore Preparation

The wild-type strain of *C. sporogenes* used in this study is ATCC15579. Isogenic mutants of this wild-type strain included: (i) a quadruple insertional knockout mutant (*gerXA^4^*^-^) lacking genes for all known GRs (Brunt et al., 2014); and (ii) mutants lacking *sleB* or *cwlJ* that were generated using the Clostron system, which inserts an erythromycin resistance cassette into the targeted gene of interest. Target sites were identified [gene; CLOSPO_00754 (*sleB*) insert site 337s and gene; CLOSPO_02089 (*cwlJ*) insert site 145s] using the Pertuka method (Perutka et al., 2004) and mutants were generated as previously described (Heap et al., 2010; Brunt et al., 2014).

Vegetative cells of *C. sporogenes* were grown anaerobically at 37°C in tryptone-yeast medium (TY) broth or on TY agar plates at 37°C. Spores of *C. sporogenes* strains were prepared in Robertson’s cooked meat broth (Southern Group Laboratories) and spores were cleaned and stored as described elsewhere (Plowman and Peck, 2002). All spores used in this study were >98% free of sporulating cells, germinated spores, and debris, as observed by phase contrast microscopy. *Escherichia coli* strains used for mutant construction were grown aerobically in Luria-Broth (LB) agar at 37°C. The *E. coli* strain Top10 (Invitrogen) was used for plasmid maintenance and the *E. coli* strain CA434 (Purdy et al., 2002) was used as the conjugation donor for plasmid DNA transfer. Where appropriate, growth media were supplemented with antibiotics or indicator reagent at the following final concentrations: chloramphenicol 25 μg mL^-1^, cycloserine 250 μg mL^-1^, thiamphenicol 15 μg mL^-1^, erythromycin 5 μg mL^-1^, and the chromogenic substrate 5-bromo-4-chloro-3-indolyl-b-D-galactopyranoside (X-Gal) 80 μg mL^-1^. All bacterial media supplements were purchased from Sigma (Gillingham, United Kingdom).

### Germinants and Spore Germination

*Clostridium sporogenes* spores (10 μL; ∼10^8^ spores mL^-1^ in water) were incubated in water for 15 min at high temperatures (70 or 80°C) and then cooled on ice before germination. Unless noted otherwise, spores were routinely heat shocked at 70°C for 15 min since treatment with 70°C gave more spore germination than with the other temperature examined in the study. For monitoring multiple individual spore’s germination using LTRS, spores were incubated at 37°C with 50 or 100 mM L-alanine in Buffer 1 [20 mM Tris-HCl (pH 7.4), 50 mM NaHCO_3_, 50 mM L-lactate]. For investigating the effects of loss of SleB, CwlJ, or GR proteins on spore germination, spores were incubated in 100 mM L-alanine in Buffer 1 at 37°C. Spore germination was also carried out with 60 mM CaDPA (made to pH 7.4 with Tris base) at 37°C and 1.0 mM dodecylamine at 45°C in 20 mM Tris-HCl (pH 7.4) and 50 mM NaHCO_3_.

In experiments measuring spores’ commitment to germinate, *C. sporogenes* spores adhered on a coverslip were exposed to 100 mM L-alanine in Buffer 1 at 37°C for 4 or 8 min, followed by germinant removal, 5 rinses with Buffer 1 by vacuum pump suction, and further incubation at 37°C in Buffer 1 for 45 min.

In experiments measuring spores’ memory of germinant stimulation, *C. sporogenes* spores adhered to a microscope coverslip were given a 4-min pulse of 100 mM L-alanine in Buffer 1 at 37°C, germination solution removed and spores rinsed five times by vacuum pump suction with 37°C Buffer 1 and held in this buffer at 37°C prior to a 2nd 4 min germinant pulse beginning at 19 or 39 min. In this experiment, spores were also germinated with 100 mM L-alanine in Buffer 1 for 8 min, followed by germinant removal and rinsing as described above and further incubation at 37°C. For quantitation of spore memory with two germinant pulses, the percentages of spores that germinated in the 2nd pulse were corrected for the percentages of spores that had germinated due to the 1st pulse.

### Phase Contrast Microscopy

Spores (1 μL; ∼10^8^ spores mL^-1^ in water) were spread on the surface of a microscope coverslip that was then dried in a vacuum desiccator for 10 min. Coverslips were suspended in water and then mounted on and sealed to a microscope sample holder held at 37°C. A phase contrast microscope was used to record the images of 500–1,000 individual spores. Spores that contain DPA appear bright and spores that do not contain DPA appear as dark. The percentages of spores containing DPA were defined as the number of bright spores divided by the total number of spores.

### Measurement of CaDPA Levels and Raman Spectra of Individual Spores by LTRS

The CaDPA levels of individual spores of various strains in water were determined by LTRS at room temperature. Briefly, an individual spore was captured with laser tweezers and its Raman spectrum was acquired with an integration time of 20 s and a laser power of 20 mW at 780 nm. Spectra of ∼50 individual spores were measured and averaged. The CaDPA level in an individual spore was determined from the peak intensity of the CaDPA-specific Raman band at 1,017 cm^-1^, which was calibrated by the peak intensity of the same Raman band from 60 mM CaDPA using the excitation volume of 1 fl to obtain attomoles of CaDPA spore^-1^, as described previously (Kong et al., 2011).

### Monitoring Germination of Multiple Individual Spores

The kinetics of CaDPA release during germination of individual *C. sporogenes* spores optically trapped by laser tweezers in liquid was measured simultaneously by Raman spectroscopy and DIC microscopy as described previously (Zhang et al., 2010b, 2014). The CaDPA level in an individual spore during germination was determined from the intensities of the CaDPA-specific Raman band at 1,017 cm^-1^, and the intensity of the DIC image was also recorded. As found previously (Zhang et al., 2010b; Kong et al., 2011), the end of the rapid fall in DIC image intensity during spore germination corresponded to the point at which release of all CaDPA was complete, and this time point was defined as *T*_release_. At this time, the DIC image intensity (*I*_release_) was 25 to 30% of that at *T*_0_, when image intensity at *T*_0_ was set at 1 and the intensity at the end of measurements was set at zero. Consequently, the CaDPA content of wild-type spores at any time relative to *T*_0_ could be estimated from the DIC image intensity, *I*_t_, as 100% × (*I*_t_ -*I*_release_)/(1 -*I*_release_), since the DIC intensity was found to be nearly coincident with the CaDPA level prior to *T*_release_ (**Figure 3B**). In addition to *T*_release_, a number of other spore germination parameters have been previously described (Zhang et al., 2010b; Kong et al., 2011; Wang et al., 2015b; Setlow et al., 2017), including: (i) *T*_1_, the time between germinant addition and initiation of a slow release of 10–20% of CaDPA which begins in some spores in populations; (ii) *T*_lag_ (termed *T*_lag1_ in the current work; see Results), the time between germinant addition and initiation of fast CaDPA release; *T*_release_, as defined above; *T*_lag2_, when the slow decrease in spores’ DIC image intensity following *T*_release_ ends (see T4 in **Figure 3B**, and in later figures); *T*_lys_, the time when spore refractility and DIC image intensity becomes constant; and Δ*T*_release_ and Δ*T*_lys_, which are calculated as *T*_release_-*T*_lag1_ and *T*_lys_-*T*_release_, respectively.

Simultaneous monitoring of the germination of multiple individual spores was by DIC microscopy as described previously (Zhang et al., 2010a,b). In brief, spores were spread on the surface of a coverslip as described above that was dried in a vacuum desiccator for ∼10 min, and coverslips were mounted on and sealed to a microscope sample holder kept at a constant temperature. The DIC images of multiple spores adhered on coverslips were recorded at a rate of 1 frame per 15 s for 30 min to 11 h by a digital charge-coupled-device camera (16 bits; 1,600 by 1,200 pixels) following the addition of preheated germinant solution to the spores on coverslips. The averaged pixel intensity of an area of 40 by 40 pixels that covered each individual spore’s DIC image was calculated, the DIC image intensity of each individual spore was plotted as a function of the incubation time with a resolution of 15 s and with initial image intensity at the first time of measurement, *T*_0_, normalized to 1, and the intensity at the end of the measurement period normally set at zero. Invariably, the DIC image intensity had been constant for ∼10 min at the end of measurements. The degree of germination of spore populations was measured by simultaneously monitoring the germination of ∼300 individual spores by DIC microscopy, and at various times the percentage of these spores that had released their CaDPA was determined as described above.

## Results

### Raman Spectra and Average CaDPA Levels of Individual *C. sporogenes* Spores

**FIGURE 1:**
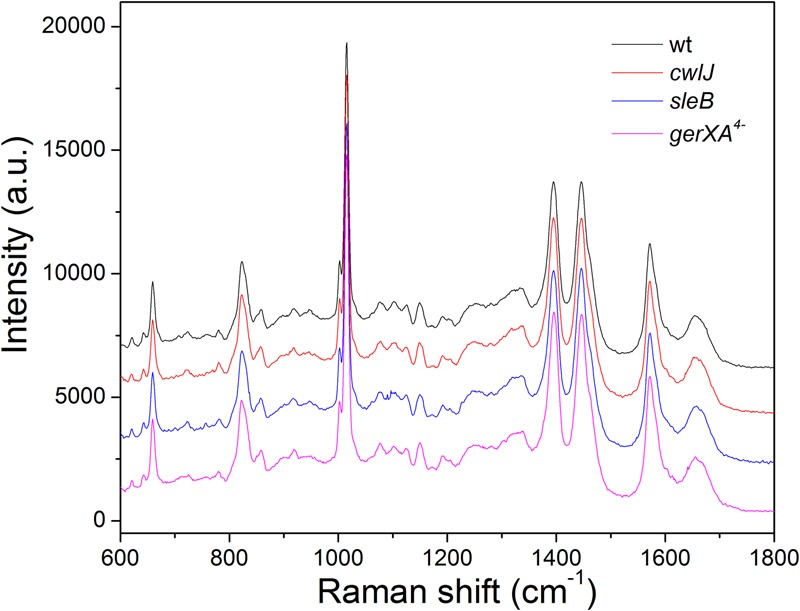
The average Raman spectra of *Clostridium sporogenes* spores of isogenic strains that retain Ca-dipicolinic acid (CaDPA). The Raman spectra of 50 individual spores of various strains were measured as described in section “Materials and Methods,” and averaged.

Laser tweezers Raman spectroscopy showed that the bands from CaDPA dominate the Raman spectra of individual *C. sporogenes* spores (**Figure 1**), just as with spores of *Bacillus* species, *C. perfringens* and *C. difficile*, as spores without CaDPA lack significant bands at 1017, 1395, and 1572 cm^-1^ (Kong et al., 2011; Wang et al., 2011, 2015c). The average intensities of the major CaDPA-specific 1,017 cm^-1^ Raman band from ∼50 individual spores in isogenic wild-type, *sleB, cwlJ*, and *gerXA^4^*^-^ strains indicated that the CaDPA levels in spores of these four strains, were 447.7 ± 45.6, 460.2 ± 35.0, 462.3 ± 50.2, and 497.4 ± 84.7 amol/spore, respectively, with no significant differences between these values in the spores of the four strains examined. These values for spore CaDPA content are similar to those in other species (Huang et al., 2007; Wang et al., 2011, 2015c).

### Effects of Heat Shock, L-Lactate and Buffers on *C. sporogenes* Spore Germination

**FIGURE 2:**
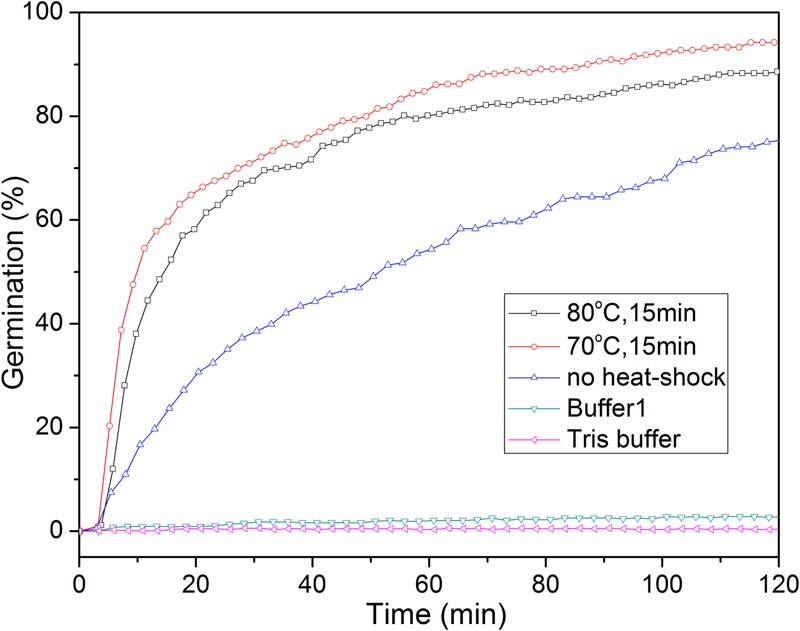
L-Alanine or buffer germination of wild-type *C. sporogenes* spores with or without with heat-activation. Spores were germinated with 100 mM L-alanine in Buffer 1 with or without prior heat shocks, or in various buffers after an optimal heat shock, and spore germination was followed by DIC microscopy, all as described in section “Materials and Methods.” Buffer 1 is 20 mM Tris-HCl (pH 7.4)/50 mM NaHCO_3_/50 mM L-lactate; and Tris buffer is 20 mM Tris-HCl (pH 7.4).

A short treatment at a high but generally sub-lethal temperature termed a heat shock synchronizes and potentiates the IM GR-dependent germination of spores of *Bacillus* and at least some *Clostridium* species (Collado et al., 2006; Zhang et al., 2009; Luu et al., 2015; Setlow et al., 2017). However, the precise mechanism of the heat shock effect is unknown (Zhang et al., 2009). A heat shock also stimulated the L-alanine germination of *C. sporogenes* spores, and this germination is GR-dependent [(Brunt et al., 2014); and see below]. Heat shock at 70°C for 15 min had a stronger effect on the extent and rate of germination with L-alanine than that at 80°C for 15 min or no heat shock (**Figure 2**). Consequently, unless noted otherwise, all spores used for L-alanine germination in this work were heat shocked at 70°C for 15 min.

Previous reports showed that L-lactate could stimulate *C. sporogenes* spore germination with some amino acids, although alone triggered no spore germination (Brunt et al., 2014). We also found that 50 mM L-lactate/20 mM Tris-HCl (pH 7.4)/50 mM NaHCO_3_ (Buffer 1) or 20 mM Tris-HCl (pH 7.4) did not trigger *C. sporogenes* spore germination (<0.3% with 300 spores examined) (**Figure 2**), consistent with the previous work (Brunt et al., 2014). However, we routinely added L-lactate/NaHCO_3_ to Tris-HCl buffer in L-alanine germinations, in case we later had to test spore germination by other amino acids.

### Kinetics of Germination of Individual *C. sporogenes* Spores with L-Alanine

**FIGURE 3:**
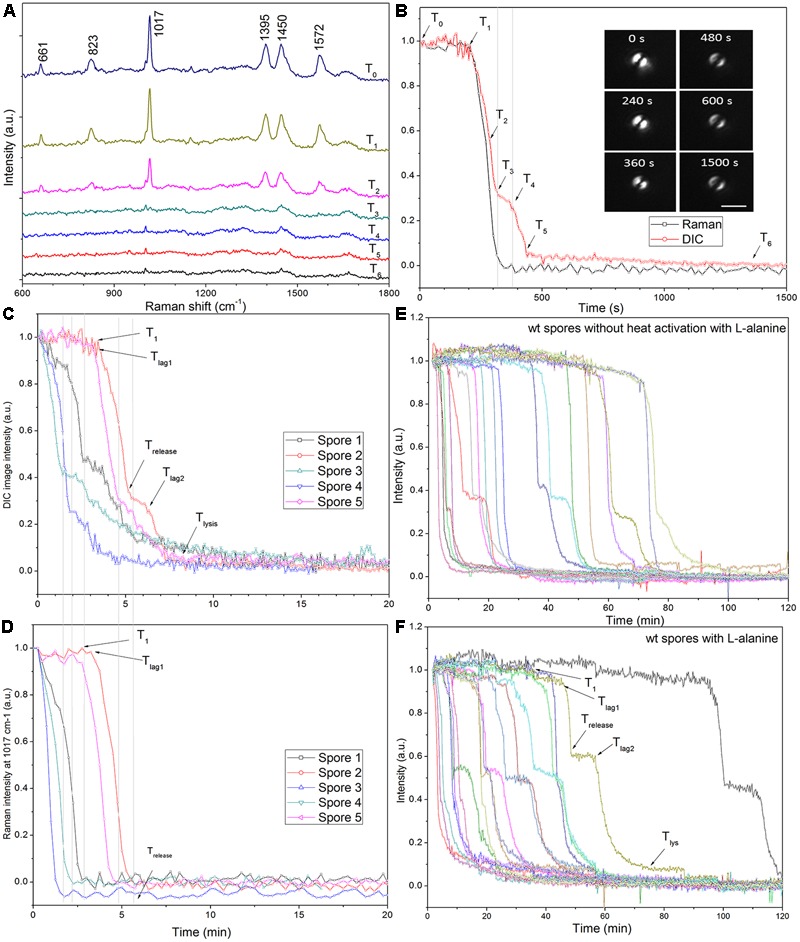
Germination of individual optically trapped wild-type *C. sporogenes* spores monitored by Raman spectroscopy and/or DIC microscopy. **(A)** Time-lapse Raman spectra of a single optically trapped spore germinating with 50 mM L-alanine as described in Methods. **(B)** The peak intensities of the 1017 cm^-1^ CaDPA-specific Raman band and the DIC image intensities are plotted vs. incubation time and DIC images taken at various times are also shown. Note that the spore appears as a double image due to the differential interference of the illumination polarized light. The laser power was 2.5 mW, Raman acquisition was 15 s per spectrum, and DIC image acquisition was 5 s per image. **(C,D)** The DIC image intensity **(C)** and the intensity of the CaDPA-specific Raman band at 1017 cm^-1^ of five individual spores germinating with 50 mM L-alanine are shown. **(E,F)** Germination was with 100 mM L-alanine without **(E)** or with **(A–D,F)** optimal heat activation and was measured by DIC microscopy, all as described in section “Materials and Methods.” In the experiments shown in **(E,F)** the level of germination after 120 min was measured by examination of 300 individual spores, and was: **(E)** 75%; and **(F)** 95%. In all panels, the DIC image and Raman band intensities in arbitrary units (a.u.) were normalized to 1 based on the respective values at the first time of measurement, and DIC image intensities at the end of the experiment were set at 0.

Laser tweezers Raman spectroscopy and DIC microscopy were used to analyze the dynamic germination of individual *C. sporogenes* spores (**Figure 3**). The results with a single optically trapped *C. sporogenes* wild-type spore germinating with 100 mM L-alanine indicated that with some spores, beginning at *T*_1_ there was an initial period of slower CaDPA release (**Figures 3C,D**) preceding the rapid fall in the spore’s DIC image intensity beginning at *T*_lag1_ and ending at *T*_release_. This fast DIC image intensity decrease closely paralleled the fast release of CaDPA seen in the spore’s time lapse Raman spectra (**Figures 3A,B**). The slower initial CaDPA release (5–10% of all CaDPA) was often difficult to see in spores germinating rapidly, but was more obvious in spores germinating more slowly (**Figures 3E,F**). Single spores followed by LTRS also exhibited a further decrease in their DIC image intensity (20–40% of the total) between *T*_release_ and *T*_lys_ (**Figures 3C–F**), presumably due to cortex hydrolysis and core swelling and water uptake. Indeed, for at least one spore, significant DIC image intensity was retained until until *T*_5_, long after all CaDPA had been released (**Figure 3B**). These observations were also made upon examination of changes in the CaDPA and DIC image intensity of more individual spores germinating with L-alanine (**Figures 3C–F**). However, following *T*_release_, some of these spores exhibited a slow decrease in DIC image intensity which was then followed by a faster decrease in ending at *T*_lys_ (**Figure 3C**). With these latter spores, the end of the pause or the slow decline in DIC image intensity between *T*_release_ and *T*_lys_ in the L-alanine germination of C. *sporogenes* spore was termed *T*_lag2_, and therefore *T*_lag_ was routinely termed *T*_lag1_ (**Figure 3C**). Given the good correspondence between CaDPA release and loss of DIC image intensity in spore germination, as in previous studies with *B. subtilis* spores (Zhang et al., 2010b; Kong et al., 2011), DIC microscopy alone was used to monitor the germination of multiple individual *C. sporogenes* spores in further experiments. The two phases in the decline in DIC image intensity following *T*_release_ during L-alanine germination were more accurately quantitated when L-alanine germination of ∼300 individual spores with or without heat shock was followed by DIC microscopy (**Figures 3E,F** and **Table 1**). In these experiments, of the spores germinating with or without an optimal heat shock, 35 and 22%, respectively, showed an obvious *T*_lag2_ inflection. Notably, while the average values of times for fast release of CaDPA (Δ*T*_release_) in the L-alanine germination of multiple individual spores were similar with spores with and without a heat shock, optimally heat-shocked spores showed the lowest values of *T*_1_, *T*_lag1_, *T*_release_, and *T*_lys_, (**Table 1**). These results suggest that the primary effect of an optimal heat shock on *C. sporogenes* spore germination is to shorten the *T*_1_ values for individual spores, as seen with spores of other species (Yi and Setlow, 2010; Zhang et al., 2010a; Byun et al., 2011; Setlow et al., 2017).

**Table 1:** Mean values and standard deviations of kinetic parameters of *Clostridium sporogenes* wild-type spore germination with various germinants and with various heat shocks^∗^.

Treatment	*T*_1_ (min)	*T*_lag1_ (min)	Δ*T*_leakage_ (min)	*T*_release_ (min)	Δ*T*_release_ (min)	*T*_lys_ (min)	Δ*T*_lys_ (min)	Percent of spores that show a *T*_lag2_	Percent germination in 2 h (number of germinated spores)
L-alanine, 70°C 15 min	5.6 ± 6.2	8.6 ± 12.5	3.0 ± 8.3	12.1 ± 12.5	3.5 ± 1.5	27.3 ± 15.0	15.2 ± 6.5	35.2%	94.5% (313)
L-alanine, 80°C 15 min	9.9 ± 11.7	17.0 ± 22.9	7.1 ± 12.7	21.7 ± 22.6	3.7 ± 2.2	45.7 ± 22.4	24.0 ± 11.8	59.0%	86.4% (218)
L-alanine, No heat shock	20.5 ± 20.2	34.8 ± 30.0	14.3 ± 16.7	38.2 ± 30.1	3.4 ± 1.3	61.3 ± 25.6	23.1 ± 9.4	21.6%	75.4% (228)
Dda	10.5 ± 13.5	16.5 ± 17.6	6.1 ± 13.7	18.8 ± 17.5	2.3 ± 1.0	35.6 ± 19.5	16.7 ± 12.5	0	48.6% (252)
CaDPA	14.3 ± 13.5	19.5 ± 14.2	5.1 ± 5.5	22.5 ± 14.2	3.0 ± 1.0	34.3 ± 15.5	11.7 ± 8.1	15.7%	90.1% (570)

*^∗^Wild-type *C. sporogenes* spores without or with heat-activation conditions as indicated were germinated as described in section “Materials and Methods” with 100 mM *L*-alanine, dodecylamine (Dda) or CaDPA, the germination of >250 individual spores followed by DIC microscopy, and kinetic parameters of spore germination were calculated, all as described in section “Materials and Methods.”*

### Germination of *C. sporogenes* Spores Lacking GRs and CLES with L-Alanine

**FIGURE 4:**
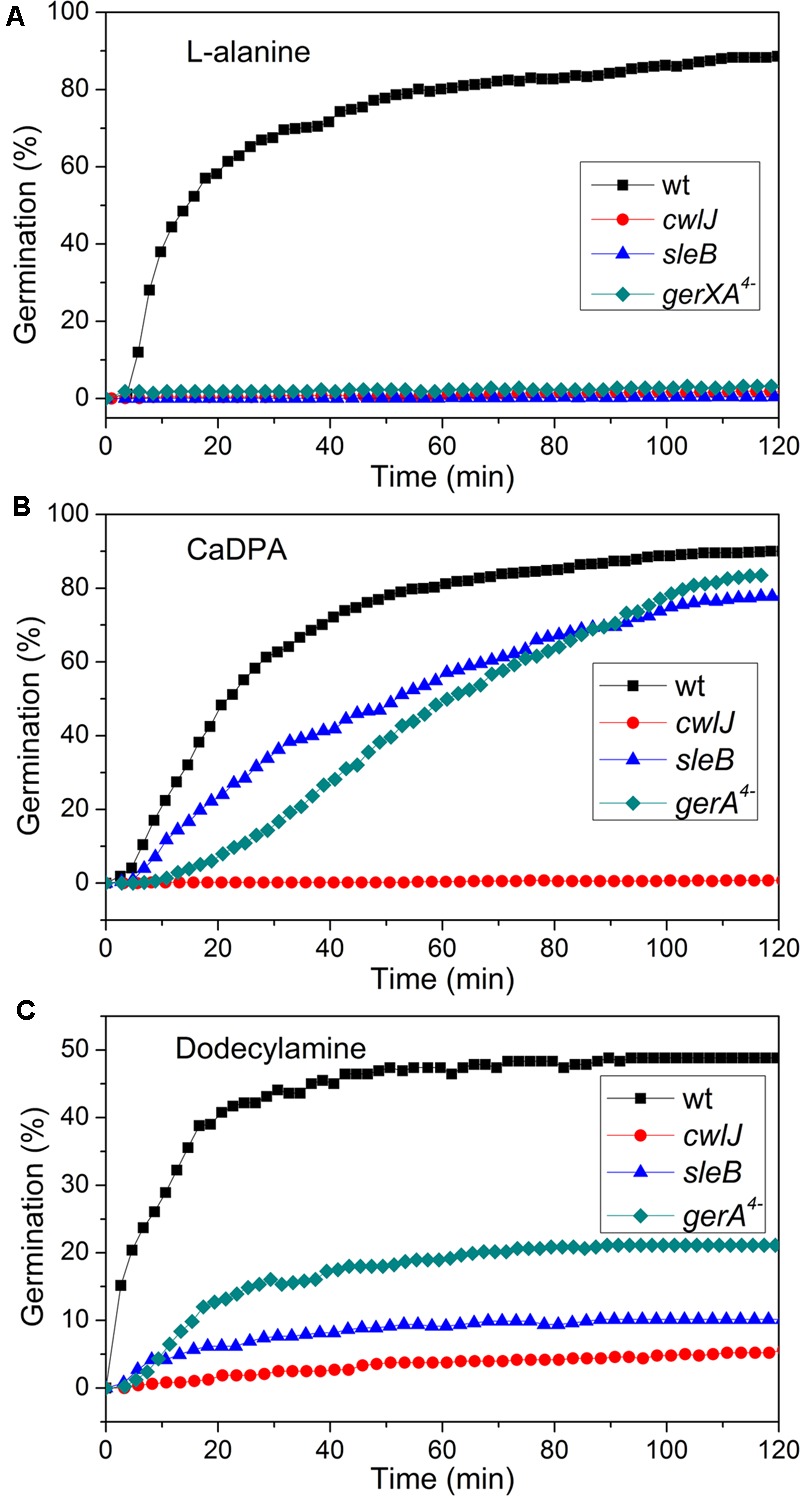
Germination of *C. sporogenes* spores of strains with or without mutations eliminating germination proteins with different germinants. Germinations were with: **(A)** 100 mM L-alanine after optimal heat shock; **(B)** CaDPA; and **(C)** dodecylamine, all as described in section “Materials and Methods,” and germination was assessed by monitoring the DIC image intensities of 300 randomly chosen individual spores.

**FIGURE 5:**
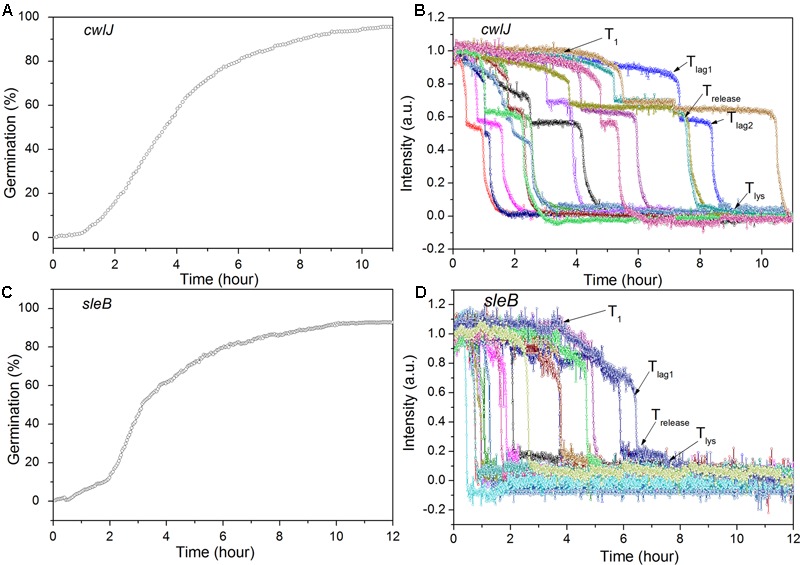
Germination of multiple individual *C. sporogenes cwlJ* **(A,B)** and *sleB* **(C,D)** optimally heat shocked spores with L-alanine over 12 h. Spores were germinated with 100 mM L-alanine for ∼12 h and germination of multiple individual spores was followed by DIC microscopy, with data from ≥300 spores for the curves in **(A,C)**, and for 15 individual spores in **(B,D)**, all as described in section “Materials and Methods.”

Previous work has found that *C. sporogenes* spores lacking most GR proteins exhibit minimal germination with L-alanine over many hours (Brunt et al., 2014). This was also found in the current work, as the *gerXA^4^*^-^ spores exhibited ≤1% germination in 2 h, and ≤1% germination even in 12 h (**Figure 4A**; and data not shown), consistent with the absence of all functional GRs and the tremendously reduced viability of *gerXA^4^*^-^ spores (Brunt et al., 2014). In *Bacillus* species, two CLEs, SleB, and CwlJ, play redundant roles in spore germination as either protein alone can give full cortex hydrolysis, and CaDPA release is only slightly slowed in *cwlJ sleB* spores (Setlow et al., 2017). SleB appears to be a lytic transglycosylase that is synthesized in the forespore and is present in spores in a mature form; this is likely also the case for CwlJ, although its bond specificity is not clear (Moriyama et al., 1996, 1999; Setlow et al., 2017). Surprisingly, the absence of either CwlJ or SleB greatly reduced *C. sporogenes* spore germination with L-alanine, as there was ≤2% germination in 2 h (**Figure 4A**). However, if the L-alanine germination time was followed for 12 h, both *cwlJ* and *sleB* spores exhibited >90% spore germination (**Figures 5A,C**). Notably, almost all germinating *cwlJ* spores examined exhibited obvious *T*_lag2_ times, while germinating *sleB* spores did not (**Figures 5B,D** and **Table 2**). Although almost all average times of various events in the L-alanine germination of individual *cwlJ* and *sleB C. sporogenes* spores were much larger than with wild-type spores under the same germination conditions, the average value of Δ*T*_release_ increased only ∼ 2-fold, and only with *sleB* spores (**Tables 1, 2**).

**Table 2:** Mean values and standard deviations of parameters of mutant *C. sporogenes* spore germination with L-alanine, dodecylamine or CaDPA^∗^.

Strain and germinant	*T*_1_ (min)	*T*_lag1_ (min)	Δ*T*_leakage_ (min)	*T*_release_ (min)	Δ*T*_release_ (min)	*T*_lag2_ (min)	*T*_lag2_–*T*_release_ (min)	*T*_lys_ (min)	*T*_lys_–*T*_lag2_ (min)	Percent of spores that show a *T*_lag2_	Percent germination in 2 h or 12 h (number of germinated spores)
^a^*cwlJ* L-alanine	145.2 ± 84.6	192.9 ± 92.3	47.7 ± 43.8	197.5 ± 91.6	4.6 ± 3.3	265.9 ± 109.0	68.4 ± 44.7	312.1 ± 107.8	46.2 ± 14.0	95.5%	95.5% (888)
*cwlJ* Dda	26.9 ± 29.5	40.1 ± 33.3	13.2 ± 13.2	42.5 ± 33.2	2.4 ± 0.9	52.3 ± 38.3	9.8 ± 11.2	63.5 ± 32.0	11.1 ± 6.4	5.6%	5.6% (26)
*^a^sleB* L-alanine	139.6 ± 109.2	219.9 ± 147.5	79.1 ± 183	228.8 ± 147.6	8.9 ± 3.4	NA	NA	267.1 ± 152.9	38.3 ± 30.1	0	92.1 (460)
*sleB* Dda	18.5 ± 31.0	29.9± 42.3	11.4 ± 19.0	34.4 ± 42.7	4.5 ± 2.7	NA	NA	64.6 ± 55.3	30.1 ± 30.8	0	11.6% (46)
*sleB* CaDPA	27.8 ± 26.4	41.7 ± 31.3	13.9 ± 18.7	44.9 ± 31.5	3.2 ± 0.9	NA	NA	79.6 ± 34.7	34.7 ± 16.0	0	80% (370)
*gerXA^4^*^-^Dda	15.9 ± 16.3	18.2 ± 17.6	2.3 ± 2.8	19.3 ± 17.4	1.1 ± 1.6	NA	NA	37.7 ± 22.4	18.4 ± 17.0	0	21.1% (88)
*gerXA^4^*^-^CaDPA	51.2 ± 25.7	53.0 ± 26.0	1.8 ± 7.5	53.6 ± 25.9	0.6 ± 0.5	NA	NA	57.6 ± 26.1	3.9 ± 7.3	0	83.5% (435)

*^∗^Clostridium sporogenes cwlJ, sleB and *gerXA^*4*-^* spores were germinated as described in the section “Materials and Methods” with 100 mM *L*-alanine, dodecylamine (Dda) or CaDPA, the germination of >400 individual spores followed by DIC microscopy, and kinetic parameters of spore germination were calculated, all as described in section “Materials and Methods.” ^a^For *cwlJ* and *sleB* spore germination with L-alanine, the experiments were for 12 h. NA, not applicable.*

### Germination of *C. sporogenes* Wild-Type and Mutant Spores with CaDPA and Dodecylamine

**FIGURE 6:**
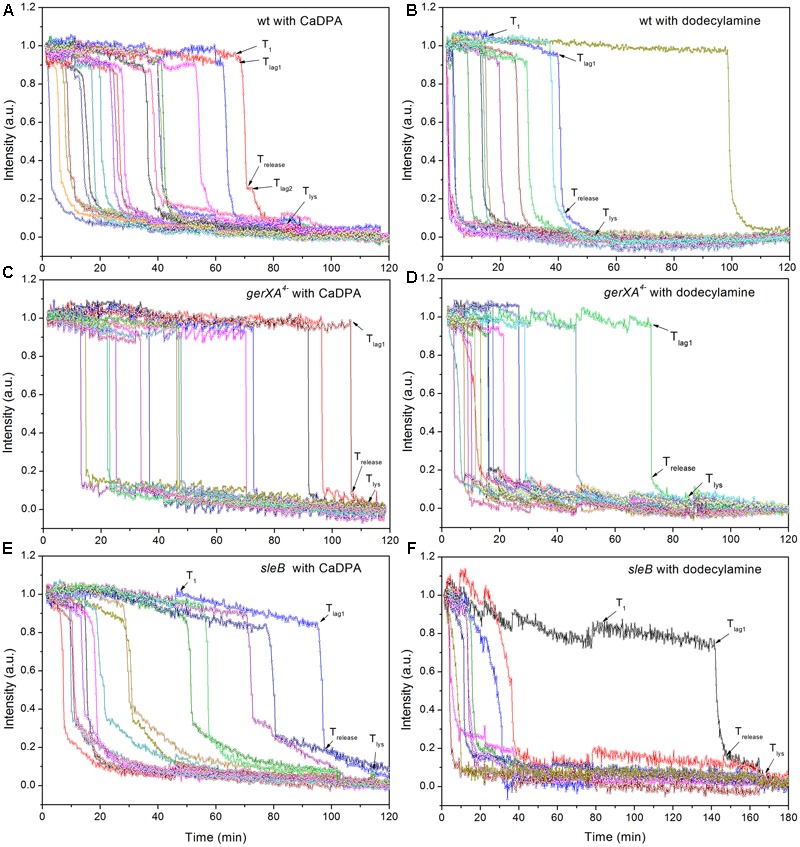
Germination of multiple individual wild-type **(A,B)**, *gerXA^4-^* **(C,D)**, and *sleB* **(E,F)** spores with CaDPA **(A,C,E)** or dodecylamine **(B,D,F)**. Spores of various strains were germinated with CaDPA or dodecylamine and germination of multiple individual spores was followed by DIC microscopy, all as described in section “Materials and Methods.”

In addition to germination stimulated by the GR-dependent germinant L-alanine, we also tested whether wild-type *C. sporogenes* spores were responsive to the GR-independent germinants CaDPA and dodecylamine that trigger germination of spores of *Bacillales* and some *Clostridiales* spores (Setlow, 2003; Olguin-Araneda et al., 2015; Wang et al., 2015c; Setlow et al., 2017) (**Figures 4B,C**). Wild-type *C. sporogenes* spores germinated >90% in 60 mM CaDPA in 2 h, but only ∼50% with 1 mM dodecylamine, and this value had become level at 50 min. Compared to germination with L-alanine, analysis of the germination of multiple individual wild-type *C. sporogenes* spores with dodecylamine (**Figures 6A,B**) gave a smaller average Δ*T*_release_ time, larger average *T*_1_, *T*_lag1_, *T*_release_, and *T*_lys_ times and similar average Δ*T*_lys_ times; germination with CaDPA had larger average *T*_lag1_, *T*_release_ and *T*_lys_ times and smaller average Δ*T*_release_ and Δ*T*_lys_ values (**Table 1**). In addition, 16% of wild-type spores germinating with CaDPA showed *T*_lag2_ times following *T*_release_, but minimal if any wild-type spores germinating with dodecylamine exhibited a *T*_lag2_ (**Figures 6A,B** and **Table 1**).

CaDPA and dodecylamine also triggered the germination of *gerXA^4^*^-^ and *sleB* spores, albeit to a lesser extent than with wild-type spores (**Figures 4B,C, 6C–F**). Minimal if any of these two mutant spores exhibited *T*_lag2_ times in germination with CaDPA and dodecylamine, and average times for various germination events were otherwise relatively similar between wild-type and *gerXA^4^*^-^ spores but higher for *sleB* spores (**Tables 1, 2**). Dodecylamine also germinated *cwlJ* spores, and with larger germination time parameters than with wild-type spores, and a very low percentage of spores with a *T*_lag2_ (**Figure 4C** and **Table 2**). However, CaDPA gave minimal germination of *cwlJ* spores in 2 h (<5%), consistent with CwlJ being activated by CaDPA as is the case in spores of *Bacillus* species.

### Commitment and Memory of *C. sporogenes* Spores in L-Alanine Germination

**FIGURE 7:**
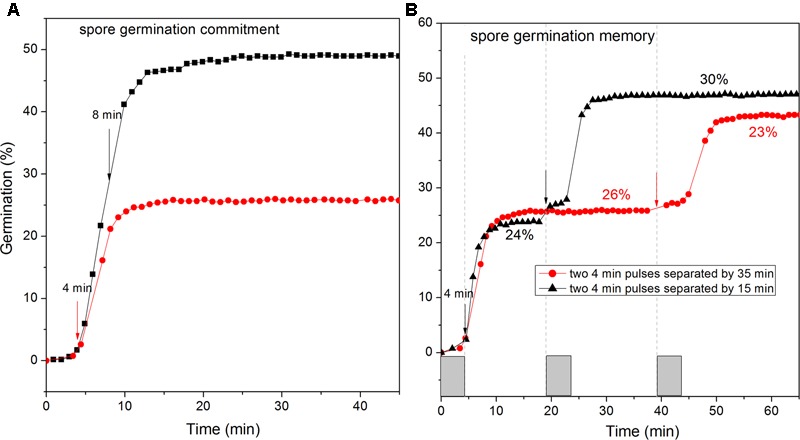
Commitment **(A)** and memory **(B)** of wild-type *C. sporogenes* spores in L-alanine germination. **(A)** Heat-activated spores were germinated with 100 mM L-alanine for 4 min or 8 min (vertical arrows denote ends of germinant exposures), followed by germinant removal, rinsing with vacuum suction, and further incubation at 37°C in Buffer 1, and germination of >300 spores was followed by DIC microscopy, all as described in Methods. **(B)** As described in section “Materials and Methods,” *C. sporogenes* spores were given a 4 min pulse of 100 mM L-alanine at *t*_0_, and then rinsed by vacuum suction and given a 2nd L-alanine pulse at 19 or 39 min, rinsed again and incubated further, and completion of spore germination of >300 spores was measured by DIC microscopy. Note that values for the % germination after the 2nd pulse was completed have been corrected for the % germination after the 1st pulse.

A notable feature of *Bacillus* and *C. difficile* spores’ germination is their irreversible commitment to germination, followed by CaDPA release after a short GR-dependent exposure (Yi and Setlow, 2010; Luu and Setlow, 2014; Wang et al., 2015b). Thus we examined if *C. sporogenes* spores also showed commitment after brief exposures to L-alanine, followed by germinant removal, extensive rinsing, and further incubation in buffer. Like *Bacillus* and *C. difficile* spores, *C. sporogenes* spores given a short germinant exposure continued to release CaDPA after germinant removal. Indeed, *C. sporogenes* spores given 4 or 8 min pulses of L-alanine that gave only 3 and 22% germination, respectively, at the time of germinant removal ultimately released 26 and 50% of their CaDPA, respectively (**Figure 7A**). These results suggest that *C. sporogenes* spores also commit to germination shortly after germinant exposure.

A recent novel finding in spore germination is that spores of at least several *Bacillus* species as well as *C. difficile* exhibit memory of germinant stimuli such that spores given a 1st germinant pulse exhibit greater germination after a 2nd pulse soon after the 1st one; however, this memory decays quickly when the separation between the two pulses is increased (Wang et al., 2015a). With *C. sporogenes* spores given a 4 min L-alanine pulse, there was ∼24% germination which ended at ∼10 min (**Figure 7B**). If a 2nd 4 min L-alanine pulse was then given at 19 min, ∼30% of the remaining dormant spores germinated, but if the 2nd pulse was not given until 39 min, only 23% of the spores remaining dormant after the 1st pulse germinated after the 2nd pulse (**Figure 7B**). However, this difference of 7% was small, and not as large that in *B. subtilis* spores germination memory, indicating that *C. sporogenes* spore memory in GR-dependent germination is low.

## Discussion

In many respects, the germination of multiple individual *C. sporogenes* spores is similar to that of individual spores of other Bacillales and Clostridiales species that have been studied (Setlow et al., 2017). In particular, there is great heterogeneity between individual spores in populations in their germination, with some spore germinating very rapidly and some not for many h. As seen previously, most of this heterogeneity is in the time, *T*_1_, when initiation of slow CaDPA leakage begins prior to the initiation of fast CaDPA release at a time designated *T*_lag_ for spores of other species, but *T*_lag1_ for *C. sporogenes* spores to distinguish it from *T*_lag2_ period between *T*_release_ and *T*_lys_. In spores of at least *B. subtilis*, one cause of spore germination heterogeneity with GR-dependent germinants is variable numbers of GRs between individuals in spore populations (Setlow et al., 2017), but there are no data on this point with *C. sporogenes* spores. It is likely that the *T*_1_ point in germination is also when spores become committed to germinate even if germinants are removed (Luu and Setlow, 2014; Setlow et al., 2017), and as shown in the present work, *C. sporogenes* spores do exhibit commitment to germinate. However, while there is leakage of ∼20% of CaDPA prior to *T*_lag_ in spores of other species (Wang et al., 2011, 2015b,c; Setlow et al., 2017), there was only 5–10% CaDPA leakage between *T*_1_ and *T*_lag1_ during wild-type *C. sporogenes* spore germination, and for some spores the *T*_1_ point was difficult to identify.

Like GR-dependent germination of spores of Bacillales species and *C. perfringens, C. sporogenes* spore germination with GR-dependent germinants was also stimulated significantly by a heat shock (Wang et al., 2011; Luu et al., 2015; Setlow et al., 2017). Notably, this treatment significantly decreases *T*_l_ values, as it did with *C. sporogenes* spores germinating with L-alanine. However, *C. sporogenes* spores with ∼4-fold differences in *T*_1_ values exhibited almost identical values of Δ*T*_release_ and Δ*T*_lys_ in L-alanine germination, again strongly indicating that *C. sporogenes* spore germination heterogeneity is primarily because of variable *T*_1_ times (and in some cases perhaps *T*_lag1_ times), and with less variability in the time needed for later germination events. Notably with spores of *B. subtilis*, germination by at least one germinant mixture is stimulated much more than fourfold by optimal heat activation (Luu et al., 2015).

As expected *C. sporogenes* spores germinated with both CaDPA and dodecylamine. In *Bacillus* spores CaDPA germination is via activation of CwlJ, although in *C. perfringens* spores that lack CwlJ and have SleC, CaDPA germination may be via stimulation of GRs; notably *C. difficile* spores that have neither CwJ nor IM GRs do not germinate with CaDPA (Wang et al., 2015c; Setlow et al., 2017). In contrast, dodecylamine germination in spores of Bacillales species and *C. difficile* appears to be via activation of the SpoVA protein channel for CaDPA release, although there may be some inner membrane GR involvement in dodecylamine germination of spores of *C. perfringens* (Wang et al., 2011, 2015c; Velásquez et al., 2014; Setlow et al., 2017). With *C. sporogenes* spores, CaDPA and dodecylamine germination were slightly slower than optimal L-alanine germination, and with dodecylamine was less complete, but Δ*T*_release_ and Δ*T*_lys_ values were similar to those for optimal L-alanine germination. Surprisingly *gerXA*^4-^ spore germination with CaDPA was significantly slower than wild-type spores as reflected in higher average *T*_1_ values. Dodecylamine germination of *gerXA*^4-^ spores exhibited a similar *T*_1_ value to wild-type spores, but many fewer spores germinated, and this was also true for *cwlJ* spores. As expected *cwlJ* spores did not germinate with CaDPA.

Importantly, the fall in *C. sporogenes* wild-type spores’ DIC image intensity between *T*_release_ and *T*_lys_ during dodecylamine germination was essentially identical to that seen in L-alanine germination. This finding indicates that CaDPA release from *C. sporogenes* spores during dodecylamine germination then triggers cortex hydrolysis by either CwlJ or SleB, just as seen during germination of spores of Bacillales species (Setlow et al., 2017). However, this is in contrast to the absence of cortex hydrolysis following CaDPA release in the dodecylamine germination of spores of either *C. perfringens* or *C. difficile*, as CaDPA release from these spores does not trigger cortex lysis (Setlow et al., 2017). Thus, spores of *C. difficile* and *C. perfringens* that lack CaDPA due to loss of genes encoding DPA synthase can be readily be isolated and are stable. In contrast, CaDPA-less spores of Bacillales species are very unstable and generally germinate spontaneously either within the sporulating cell or soon after release from the sporangium. Consequently, we would expect that *C. sporogenes* spores that lack CaDPA would also be unstable and rapidly germinate spontaneously as they indeed do (Brunt et al., 2016).

As seen previously with spores of other Bacillales and Clostridiales species the GR-dependent germination of *C. sporogenes* spores exhibited at least four distinct phases in germination: (i) no relevant change between *T*_0_ and *T*_1_; (ii) slow CaDPA leakage between *T*_1_ and *T*_lag1_; (iii) fast release of all remaining CaDPA between *T*_lag1_ and *T*_release_, and finally (iv) a further decline in DIC image intensity between *T*_release_ and *T*_lys_ which is due to cortex hydrolysis and core swelling with water uptake (Setlow et al., 2017). While these periods were present in germination of most *C. sporogenes* spores, it was sometimes difficult to see the initial CaDPA leakage between *T*_1_ to *T*_lag1_, as amounts of CaDPA leakage in this period were often ≤5% of total CaDPA, generally lower than with germinating spores of other species (Wang et al., 2015b,c). Presumably *C. sporogenes* spores require much less CaDPA leakage to trigger rapid CaDPA release that do spores of other species.

The most striking novel feature of the GR-dependent germination of optimally heat activated wild-type *C. sporogenes* spores was in the *T*_release_–*T*_lys_ period when 35% of spores exhibited a second lag or slow period of decline in DIC image intensity prior to a more rapid decline to *T*_lys_, something that has not been seen in the germination of spores of other species. The reason for this pause or slowing in the decline in DIC image intensity between *T*_release_ and *T*_lys_ is not clear. However, it is notable that *sleB* spores germinating with L-alanine exhibited no *T*_lag2_, while ∼95% of *cwlJ* spores germinating with L-alanine exhibited this *T*_lag2_. This suggests that the absence of *T*_lag2_ in some spores is due to their low levels of CwlJ. Perhaps there are variable CwlJ levels in individuals in *C. sporogenes* spore populations, with some spores having such low CwlJ levels that *T*_lag2_ is observed in spore germination. Notably, a 80°C heat shock increased the percentage of spores that exhibited a *T*_lag2_ ∼3-fold over that with spores not given a heat shock, and at least in *B. subtilis* spores, CwlJ is significantly less heat stable than is SleB (Atrih and Foster, 2001); perhaps the 80°C heat shock preferentially inactivated *C. sporogenes* CwlJ. However, why CwlJ action should lead to a *T*_lag2_ is not clear. Notably, levels of *C. sporogenes* spores exhibiting a *T*_lag2_ were much lower in germination with dodecylamine, which almost certainly does not explicitly require GRs, and not only wild-type and *sleB* spores, but also with *cwlJ* spores. This suggests that there is some interaction between CLEs and GRs, primarily between CwlJ and GRs, that suppresses the *T*_lag2_ phenomenon. Indeed, with GRs absent, the frequency of *T*_lag2_ was greatly reduced, as it was in *gerXA*^4-^ spores germinating with either dodecylamine or CaDPA. That there is some type of interaction, either directly or indirectly, between GRs and CLEs is also indicated by the much slower germination of *gerXA*^4-^ spores with both CaDPA or dodecylamine. This effect was reciprocal, as the absence of either SleB or CwlJ also slowed *C. sporogenes* germination with L-alanine ∼20-fold based on *T*_1_ values, but with minimal effects if any on Δ*T*_release_, although Δ*T*_lys_ values were increased 2- to 5-fold. Previous work with *Bacillus* spores has not observed this *T*_lag2_ germination phenomenon, and the reason(s) for it in *C. sporogenes* spore germination is unclear.

The lack of CwlJ in spores of *Bacillus* species increases Δ*T*_release_ values in germination with both GR-dependent and GR-independent germinants up to 10-fold (Setlow et al., 2017), but this was not seen with *C. sporogenes* spores. Thus, it appears that there are significant differences in the relationship between CLEs and GRs in the triggering of CaDPA release in germination between *C. sporogenes* spores and those of *Bacillus* species, although how this differs at the molecular level in spores is not clear.

Overall, the work reported in this communication indicates that *C. sporogenes* spore germination exhibits many similarities to that of spores of Bacillales species that like *C. sporogenes* have CwlJ and SleB. However, there are some features unique to *C. sporogenes* spore germination, in particular the *T*_lag2_ period between *T*_release_ and *T*_lys_ which appears to be due somehow to lack of CwlJ action, the strong dependence of GR-dependent germination on both CwlJ and SleB, as well as the slowing of CaDPA and dodecylamine germination in GR deficient spores. What causes these latter phenomena are not yet clear and will require further study.

## Conclusion

As with *Bacillus* and some *Clostridium* spores, individual *C. sporogenes* spores germinating with L-alanine showed similar germination curves with heterogeneous *T*_1_, *T*_lag1_, *T*_release_, and *T*_lys_ times and relatively constant Δ*T*_release_ periods for individual spores. After *T*_release_, some spores also displayed another lag in rate of change in DIC image intensity before the start of a second obvious DIC image intensity decline at *T*_lag2_ prior to *T*_lys_. Almost all *C. sporogenes* spores lacking the cortex-lytic enzyme (CLE) CwlJ spores exhibited a *T*_lag2_ in L-alanine germination. Spores without the CLEs SleB or CwlJ exhibited greatly slowed germination with L-alanine, but spores lacking all GR proteins did not germinate with L-alanine. The absence of these various germination proteins also decreased but did not abolish germination with dodecylamine and CaDPA, but spores without CwlJ exhibited no germination with CaDPA. *C. sporogenes* spores also displayed commitment, but spore memory in GR-dependent germination is small.

## Author Contributions

Y-QL, JB, PS, MP, and SW planned the experiments. Y-QL, JB, and SW conducted the experiments, Y-QL and SW analyzed data, PS and SW wrote the manuscript. All authors revised and approved the manuscript.

## Conflict of Interest Statement

The authors declare that the research was conducted in the absence of any commercial or financial relationships that could be construed as a potential conflict of interest.
